# The effectiveness of exercise-based vestibular rehabilitation in adult patients with chronic dizziness: A systematic review

**DOI:** 10.12688/f1000research.14089.1

**Published:** 2018-03-05

**Authors:** Burak Kundakci, Anjum Sultana, Alan J Taylor, Mansour Abdullah Alshehri

**Affiliations:** 1 Academic Rheumatology, School of Medicine, University of Nottingham, Nottingham, Nottinghamshire, NG7 2RD, UK; 2 Physiotherapy and Rehabilitation, Faculty of Health Sciences, Ordu University , Ordu, Turkey; 3 Division of Physiotherapy, School of Health Sciences, University of Nottingham, Nottingham, Nottinghamshire , NG7 2RD, UK; 4 Physiotherapy Department, Faculty of Applied Medical Sciences, Umm-Al-Qura University, Mecca, 21421, Saudi Arabia

**Keywords:** Vestibular Rehabilitation, Exercise, Physiotherapy, Chronic Dizziness, Vertigo, Balance

## Abstract

**Background:** Dizziness is a non-specific term used by patients to describe several symptoms ranging from true vertigo, light headedness, disorientation or sense of imbalance. Vestibular rehabilitation (VR) is a specific form of exercise-based therapy programme aimed at alleviating the primary and secondary problems of a vestibular pathology. The aim of this study was to investigate the effectiveness of exercise-based vestibular rehabilitation in adult patients with chronic dizziness.

**Methods:** The following five databases were searched: the Cochrane Central Register of Controlled Trials (CENTRAL, the Cochrane Library), MEDLINE, PubMed, the Physiotherapy Evidence Database (PEDro) and Scopus (Elsevier). Two investigators independently reviewed all articles and a systematic review of literature was performed using the PRISMA guidelines (Preferred Reporting Items for Systematic Reviews and Meta-Analyses). The articles were included if they met the following inclusion criteria: (1) randomised controlled trial, (2) people with chronic dizziness, (3) adults aged 18 or over, (4) exercise-based VR, (5) VR exercises compared with sham or usual care, non-treatment or placebo and (6) only studies published full text in English.

**Results:** The initial search identified 304 articles, four of which met the criteria for analysis. All studies involved some form of vestibular rehabilitation, including vestibular compensation, vestibular adaptation and substitution exercises. These exercises were compared with usual medical care (three studies) or placebo eye exercise (one study). The Vertigo Symptom Scale was the most commonly used outcome measure to assess subjective perception of symptoms of dizziness (three studies). According to the PEDro scale, three studies were considered to be of high quality, and one was rated as fair.

**Conclusions:** This review suggests that exercise-based vestibular rehabilitation shows benefits for adult patients with chronic dizziness with regard to improvement in the vertigo symptom scale, fall risk, balance and emotional status.

## Introduction

Dizziness is a non-specific term used by patients to describe several symptoms ranging from true vertigo, light headedness, disorientation or sense of imbalance ^1^. It is a frequent complaint, especially in elderly patients, with a reported prevalence of dizziness of approximately 30% of older adults, affecting more women (36%) than men (22%). Importantly, its prevalence rises with age ^2^. Consequently, dizziness leads to a significant personal and health care burden. It is associated with a risk of falls, an increased level of fear and anxiety, and decreased independence in older people. In a recent study, Tinetti *et al.* ^3^ reported adverse health, functional and psychological consequences due to chronic dizziness in patients aged 72 years or older. It was concluded over one year of follow-up that chronic dizziness was associated with increased risk of falling, worsened depressive symptoms, self-reported health and decreased confidence in performing social activities.

Dizziness may be classified into four categories: vertigo, pre-syncope, disequilibrium and psycho-physiological ^1^. Vertigo means a rotatory movement either of the patient or of their surroundings. It is generally provoked by head movements, such as movements in bed like turning over or looking upwards. Vertigo suggests disturbances of the vestibular system ^4^. Pre-syncope is a medical term defining an imminent loss of consciousness. Patients may feel that they are about to faint, often accompanied by nausea or sweating ^4^. Disequilibrium refers to a sense of imbalance or unsteadiness, and being in a dark room or closing eyes generally deteriorates symptoms. It is attributed to somatosensory abnormality, visual illusions or cerebellar disorders ^4^. Psycho-physiological symptoms are a combination of sensations such as swimming, floating and rocking or a feeling of being removed from one’s body. This symptom can be created by anxiety and central disorders ^4^.

Dizziness may occur due to numerous diseases or disorders. The causes of dizziness can be grouped into otological, neurological and medical disorders. Otological causes consist of middle ear disease, unilateral peripheral vestibular dysfunction, benign paroxysmal positional vertigo (BPPV), bilateral vestibular failure, Ménière’s syndrome, and benign recurrent vertigo. Neurological causes include the 7 ^th^ cranial nerve, cranio-cervical junction, cerebellum, and cortex problems. Furthermore, dizziness is associated with general medical issues such as orthostatic hypotension, low cardiac output, hyperventilation, hypoglycaemia, anaemia and psychological extremes ^1^. Patients benefit from a full neuro-otological examination, explanation of their symptoms and appropriate reassurance. Even though medical management of dizziness depends on the diagnosis, numerous treatments such as vestibular rehabilitation (VR) ^4^, medication ^5^, cognitive behavioural therapy (CBT) ^6^, Tai Chi exercises ^7^ and surgery ^5^ have been advocated as management strategies.

Vestibular rehabilitation is a specific form of exercise-based therapy programme aimed at alleviating the primary and secondary problems of a vestibular pathology. The original protocols date back to 1946, when Cawthorne and Cooksey defined hierarchical group activities according to their difficulties to challenge the central nervous system. These exercises are mainly based on graded activities including eye, head and body movements stimulating the vestibular system ^8^. Today, specific exercises have been improved and further described in the VR therapy, with each exercise, such as habituation, adaptation and substitution, based on different physiological and behavioural rationales. Habituation exercises, also called compensatory exercises, use repetitive movements or provoking stimuli. The movements that provoke patients’ symptoms are identified and the patient performs these exercises until they no longer respond adversely to the stimuli. Adaptation exercises are repeated head and eye movements which aid the central nervous system by adapting to a loss or an alteration in vestibular system input. The head is moved to the left and right side, while the eyes are kept fixed on a stationary target. Substitution exercises organise the use of the remaining sensory inputs to aid postural control. It is aimed at the patient using an intact sensory system instead of a sensory system providing no input or error message for the central nervous system ^4^.

The management of dizziness is important issue in primary care at the systemic level ^9^. The occurrence of dizziness, in addition to impacting directly on patient health and quality of life, is associated with a large economic burden on the health care system. The total national cost of patients with dizziness visiting the emergency department (ED) in the US exceeds $4 billion annually, representing approximately 4% of total ED costs ^10^. In spite of the increase in use of VR, currently, there is a lack of literature investigating the effectiveness of exercise-based VR in adult patients with chronic dizziness. Therefore, the aim of this systematic review is to appraise the efficacy of exercise-based VR in adult patients with chronic dizziness. Previous reviews attempted to investigate either the effectiveness of VR on all types of chronic vestibular disorders ^11^ or general management of the patient with chronic dizziness ^12^. This systematic review (SR) aims to provide a summary of the evidence on the effectiveness of specifically exercise-based VR in adult patients with chronic dizziness.

## Methods

### Data sources

The literature search was performed by two independent researchers (B.K. and A.S.) for articles published in English. For the identification of relevant studies, the following databases were used from their establishment date as databases until January 2017: the Cochrane Central Register of Controlled Trials (CENTRAL, Cochrane Library), MEDLINE, PubMed, the Physiotherapy Evidence Database (PEDro) and Scopus (Elsevier). The key search terms used were “dizziness”, “chronic dizziness”, “vestibular rehabilitation”, “habituation exercise” and “adaptation exercise”, modified in terms of the glossary of each database and combined using Boolean operators. Table 1 shows the search strategy for one of the selected databases (PubMed). The titles and abstracts were examined according to the inclusion and exclusion criteria separately by two authors (B.K. and A.S.). In case of any doubt, the full text of an article was read to determine its relevance. Furthermore, reference lists of all identified articles were screened for additional relevant literature. After that, if there was a disagreement between the two authors (B.K. and A.S.), a third author (A.J.T.) was consulted.

**Table 1.** Search Strategy for PubMed.

Step	Search Terms and Methods
1	Search “Randomized Controlled Trial"[Publication Type]
2	Search “Controlled Clinical Trial”[Publication Type]
3	Search randomized[Title/Abstract]
4	Search placebo[Title/Abstract]
5	Search randomly[Title/Abstract]
6	Search trial[Title]
7	Search (((((“Randomized Controlled Trial"[Publication Type]) OR “Controlled Clinical Trial”[Publication Type]) OR randomized[Title/Abstract]) OR placebo[Title/Abstract]) OR randomly[Title/Abstract]) OR trial[Title]
8	Search elderly
9	Search aging
10	Search aged
11	Search adult
12	Search (((elderly) OR aging) OR aged) OR adult
13	Search “Dizziness”[MeSH Terms]
14	Search “Vertigo”[MeSH Terms]
15	Search dizz*
16	Search vertigo
17	Search (((“Dizziness”[MeSH Terms]) OR “Vertigo”[MeSH Terms]) OR dizz*) OR vertigo
18	Search "Vestibular Rehabilitation"[MeSH Terms]
19	Search Habituation exercise
20	Search Adaptation exercise
21	Search Cawthorne-Cooksey Exercises[MeSH Terms]
22	Search Gaze stabilization exercise[MeSH Terms]
23	Search "vestibular exercise"[MeSH Terms]
24	Search "repositioning maneuvres"
25	Search (((((("Vestibular Rehabilitation"[MeSH Terms]) OR Habituation exercise) OR Adaptation exercise) OR Cawthorne-Cooksey Exercises[MeSH Terms]) OR Gaze stabilization exercise[MeSH Terms]) OR "vestibular exercise"[MeSH Terms]) NOT "repositioning maneuvres"
26	Search (((((((((“Randomized Controlled Trial"[Publication Type]) OR “Controlled Clinical Trial”[Publication Type]) OR randomized[Title/Abstract]) OR placebo[Title/Abstract]) OR randomly[Title/Abstract]) OR trial[Title])) AND ((((elderly) OR aging) OR aged) OR adult)) AND ((((“Dizziness”[MeSH Terms]) OR “Vertigo”[MeSH Terms]) OR dizz*) OR vertigo)) AND ((((((("Vestibular Rehabilitation"[MeSH Terms]) OR Habituation exercise) OR Adaptation exercise) OR Cawthorne-Cooksey Exercises[MeSH Terms]) OR Gaze stabilization exercise[MeSH Terms]) OR "vestibular exercise"[MeSH Terms]) NOT "repositioning maneuvres")

### Data selection

The articles were included if they were based on the following inclusion criteria: (1) randomised controlled trials (RCTs), (2) people with chronic dizziness, whereby chronicity means longer than three months, or studies determined the case as “chronic” in the title, (3) adults aged 18 or over, (4) exercise-based VR, (5) VR exercises compared with sham or usual care, non-treatment or placebo and (6) only studies published full text in English. Articles with the following criteria were excluded: (1) non-randomised studies or non-controlled studies, (2) patients who have chronic dizziness due to only specific diagnosed causes, such as BPPV, migraine-associated vertigo or Ménière's disease, (3) studies including patients younger than 18, (4) passive VR (Canalith repositioning manoeuvres), (5) studies comparing the effectiveness of two different VR exercises and (6) non-English language studies.

### Data extraction

The following key data were independently extracted from the included articles by the two review authors (B.K. and A.S.). The data extracted included participant characteristics (population, number of participants, age and gender), types of intervention (intervention group and control group), types of outcome measures (primary and secondary outcomes) and the main findings. The methodological quality of the included studies and risk of bias were assessed according to the PEDro scale, which is commonly used in SRs ^13^. It was reported that the PEDro scale has valid and reliable criteria to measure the study quality of RCTs ^14^. The PEDro scale consists of a checklist of 11 questions regarding validity and interpretation of results of controlled trials and it is rated by assigning one point according to the positive answers, with the total number of positive answers scored out of 10 ^15^. The first question is not used to calculate the PEDro score as it shows the sample eligibility criteria. Higher scores on the PEDro scale demonstrates a more appropriate study design. Articles were scored independently by the two authors (B.K. and A.S.) and any discrepancies between the authors in the early stages of selection and assessment of risk of bias of the articles were resolved by consensus or if required, consultation with the third author (A.J.T.). The quality of studies was rated in accordance with the following scoring system, taken from prior SRs ^16, 17^, ≤3 low quality, 4-5 fair quality and ≥6 high quality.

## Results

### Study selection

**Figure 1.**
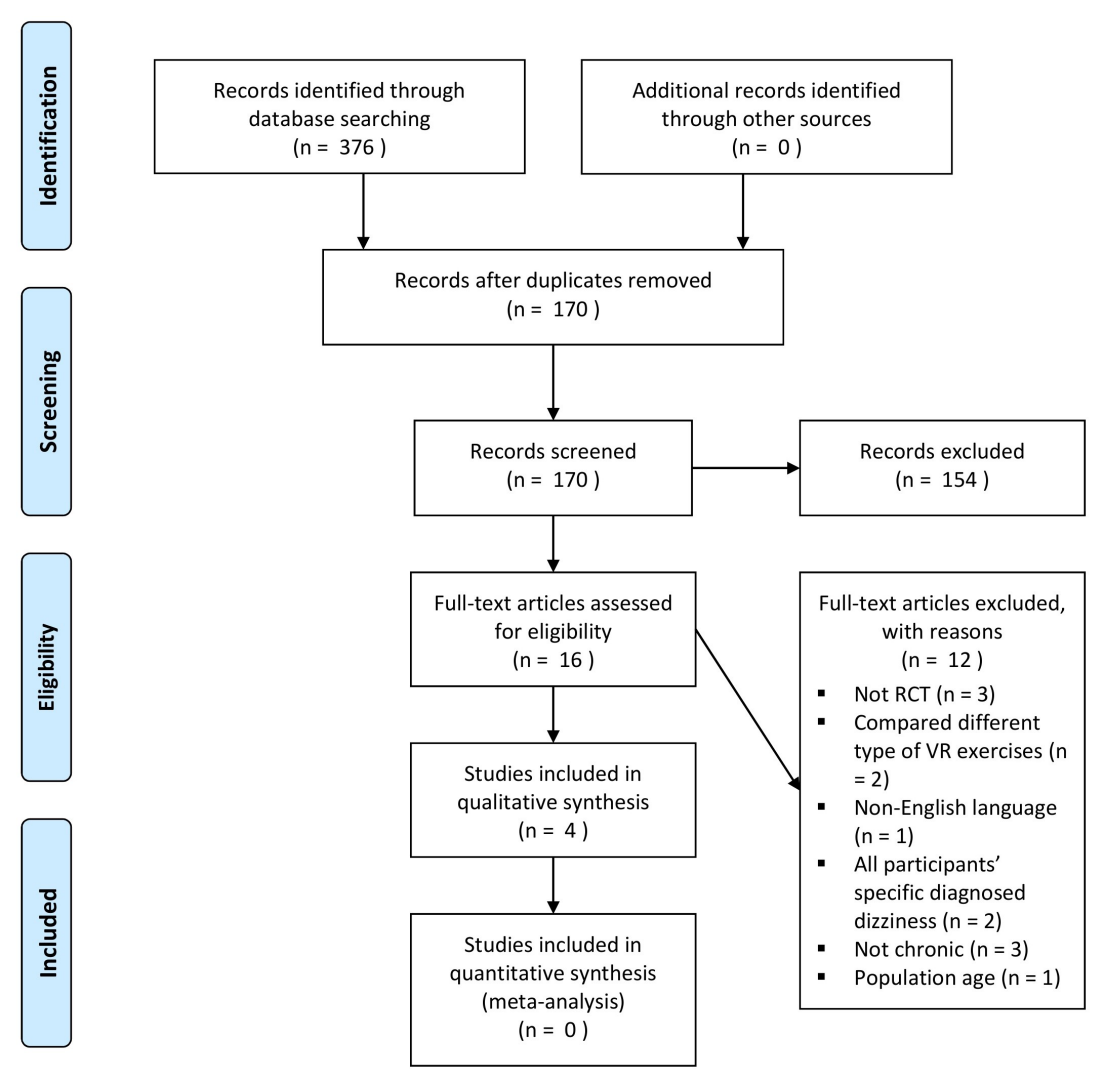
PRISMA diagram of the search strategy and study selection.

A total of 376 electronic publication records were retrieved from the systematic database search strategy. After 206 duplicates were removed, the remaining 170 articles were investigated through the title and abstract assessment and 16 records were identified as potentially eligible. After full text analysis, 12 of the remaining full text articles did not meet the inclusion criteria and were excluded because of the reasons such as language, chronicity, specific diagnosed dizziness, and lack of randomisation. As a result, a total of four articles ^18– 21^ met the inclusion criteria. The flow of studies and the reasons for exclusion are demonstrated in a PRISMA diagram ( Figure 1).

### Study characteristics

Three of the included articles originated from the UK and one was from the US. All the included studies were RCTs. A total of 687 participants were included, predominantly females (n=503). In general, the diagnosis of dizziness was non-specific, however a few causes reported included Ménière's disease, BPPV and labyrinthitis. In three studies ^18, 20, 21^, exercises were delivered under the supervision of trained nurses, and in one study ^19^, booklet-based exercises were used. All forms of VR which are compensatory, adaptation or substitution exercises were selected as the main intervention. These exercises were compared with usual medical care in three studies ^19– 21^, while they were evaluated versus placebo eye exercises in one study ^18^. The most common outcome measure was self-reported dizziness measured by the Vertigo Symptom Scale, Visual Analogue Scale or Dizziness Handicap Inventory. Other outcome measures included gait speed (Dynamic Gait Index (DGI)), balance (Postural Stability Eyes Open and Closed, Romberg Test), anxiety and depression (Hospital Anxiety and Depression Scale), quality of life (Short Form-36) and other tests (computerised dynamic posturography, customised computerised software). Table 2 describes the main study characteristics of the included studies.

**Table 2.** Main characteristics of included studies.

Study	Participants	Intervention Group(s)	Comparison Group	Outcomes	Results
Hall *et al.* ^18^	Age: above 60 years old, age (mean): intervention group (IG) = 73.6, comparison group (CG) = 74.5 Total n = 37 (9 men, 28 women) IG n = 20 (3 men, 17 women) CG n = 17 (6 men, 11 women)	The intervention group performed vestibular adaptation and substitution exercises designed to improve gaze fixation. 3 times per day, total time for gaze stability exercises did not exceed 30 minutes daily. Plus balance and gait training home exercise programme	A placebo eye exercises designed for the comparison group. Saccadic eye movements without visual targets against a white wall 3 times per day, total time did not exceed 30 minutes daily. Plus balance and gait training home exercise programme	1. Disability Rating Scale 2. Visual Analogue Scale 3. Visual acuity during head movement by using customised computerised software 4. Dynamic Gait Index (DGI) 5. Computerised dynamic posturography for sensory input	There were no significant differences between the intervention and comparison group with the exception of DGI. The intervention group showed a significant decrease in fall risk. While 90% of the intervention group showed an improvement in fall risk, in the comparison group it was 50%.
Yardley *et al.* ^19^	Age: above 18 years old, age (mean): IG = 60, CG = 58.2 Total n = 337 (98 men, 239 women) IG with telephone support, n = 112 (30 men, 82 women) IG without telephone support n = 113 (40 men, 73 women) CG n = 112 (28 men, 84 women)	Booklet based VR only and booklet based VR with telephone support Daily exercises at home for up to twelve weeks Telephone support, up to three brief sessions from a vestibular therapist	Routine care	Primary Outcome Measure 1. Vertigo symptom Scale Secondary Outcome Measures 1. Total healthcare cost per quality adjusted life year (QALY) 2. Patients reporting subjective improvement 3. Vertigo Balance Scale 4. Dizziness Handicap Inventory 5. Hospital Anxiety and depression scale	At 12 weeks, the treatment and comparison groups did not show any significant difference on the vertigo symptom scale. After one year follow-up there was a significant improvement in the intervention groups compared to the comparison group.
Yardley *et al.* ^20^	Age: above 18 years old, age (mean): IG = 60.1, CG = 59.6 Total n = 143 (28 men, 115 women) IG n = 67 (15 men, 52 women) CG n = 76 (13 men, 63 women)	30–40 minute Vestibular Compensation Exercises after assessment at baseline and 6-week follow- up Eight sets of standard head and body movements performed twice daily	Standard Medical Care	1. Vertigo Symptom Scale 2. Hospital Anxiety and Depression Scale 3. Vertigo Handicap Questionnaire 4. Provocative Movements 5. Sharpened Romberg Tests	The intervention group improved on all measures, while the comparison group demonstrated no improvement.
Yardley *et al.* ^21^	Age (mean): IG = 62.93, CG = 61.01 Total n = 170 (49 men, 121 women) IG n = 83 (24 men, 59 women) CG n = 87 (25 men, 62 women)	Nurse-delivered VR exercises. Patients were seen individually for 30 to 40 minutes to take them the booklet and additional support, after first session advice by telephone at one and three weeks	Usual medical care	Primary Outcome Measures 1. Vertigo Symptom Scale 2. Movement-Provoked Dizziness 3. Postural Stability Eyes Open and Closed 4. Dizziness Handicap Inventory Secondary Outcome Measures 1. Short Form-36 (physical functioning) 2. Hospital Anxiety and Depression Scale	There was a greater improvement on all primary outcome measures in the treatment group compared to the usual medical care.

IG - Intervention Group; CG - Control Group; n - Number; DGI - Dynamic Gait Index; VR - Vestibular Rehabilitation; QALY - Quality Adjusted Life Year.

### Risk of bias

As mentioned before, the methodological quality of the included studies and risk of bias were evaluated by using the PEDro criteria, whereby the greater the PEDro score, the more convenient the study design. Consequently, according to the PEDro assessment conducted by the authors (B.K. and A.S.), the results demonstrated that three studies were rated as high quality, and one was fair quality. Table 3 shows the quality appraisal of included studies in detail. A lower scoring criteria amongst studies also indicates more risk of bias. The common criteria that the included articles failed to meet were allocation concealment, blinding of participants’ therapists or outcome assessors in accordance with the PEDro scale. Hence, the highest risk of bias for these articles might be associated with selection and detection.

**Table 3.** Quality appraisal of included studies.

Items	Hall *et al.* ^18^	Yardley *et al.* ^19^	Yardley *et al.* ^20^	Yardley *et al.* ^21^	Total
Inclusion criteria	Y	Y	Y	Y	**4**
Random allocation	Y	Y	Y	Y	**4**
Concealed allocation	N	N	N	Y	**1**
Group similarity at baseline	Y	N	Y	Y	**3**
Blinding of participants	Y	N	N	N	**1**
Blinding of therapists	N	N	N	N	**0**
Blinding of assessors	N	Y	N	Y	**2**
Outcome measures in 85% of sample	Y	Y	Y	Y	**4**
Intention-to-treat analysis	N	Y	N	Y	**2**
Comparison between groups	Y	Y	Y	Y	**4**
Measures of central tendency and dispersion	Y	Y	Y	Y	**4**
Total score	**6**	**6**	**5**	**8**

### Data synthesis and analysis

All studies in this review differ in outcome measures used, comparator interventions or delivery method. Even though a meta-analysis was planned, it was not possible to perform it due to heterogeneity. Hence a descriptive, narrative synthesis of the main findings and quality of the included articles will be presented in this SR.

### Results of individual studies

The main findings of each study can be found in Table 2. Only one study ^18^ did not present confidence intervals (CI) for between group differences. The other three studies ^19– 21^ reported confidence intervals for the within group change scores.

### A. Changes in symptoms

All the included studies ^18– 21^ assessed whether there was an improvement in dizziness symptoms, as measured by the Vertigo Symptom Scale ^19– 21^, the Visual Analogue Scale (VAS) ^18^ or the Disability Rating Scale ^18^. Three studies ^19– 21^ reported that participants treated with VR demonstrated statistically significant improvement compared with participants treated with usual medical care. However, the study of Hall *et al.* ^18^ did not detect any significant change in the dizziness VAS score.; the magnitude of change in the dizziness VAS score was small for the gaze stabilisation group, and moderate for the placebo eye exercise group (standardised response mean (SRM) = 0.4 and 0.7, respectively).

### B. Changes in balance and risk of falls

Balance and risk of falls were investigated by the Romberg Test ^19– 21^, Vertigo Balance Scale ^19^, Dynamic Gait Index ^18^ and computerised dynamic posturography ^18^. All studies found significant differences in balance and risk of falls, with the improvement in all balance and risk of falls tests being significantly greater in the VR group compared to the routine medical care.

### C. Changes in emotional status

Anxiety and depression were evaluated using Anxiety and Depression Scale ^19– 21^, Geriatric Depression Scale or State-Trait Anxiety Inventory ^18^. While three studies ^19– 21^ demonstrated significant improvement in anxiety and depression, Hall *et al.* ^18^ could not find any significant difference.

## Discussion

The use of exercise-based VR has been investigated in terms of its ability to manage adult patients with chronic dizziness. The literature search confirmed the dearth of RCTs evaluating the efficacy of VR. However, despite the scarcity of studies, the articles included in this SR suggested that VR might be an area for future research with regard to severity of dizziness, fall risk, handicap, postural control and emotional status. However, owing to the differences in the methodologies of included studies, it was not possible to determine the most effective VR protocol, or the ideal frequency and intensity of the interventions.

In this context, the databases mentioned previously were scrutinised to identify relevant literature. Four studies ^18– 21^ were found to be eligible in terms of participant characteristics, study design, interventions and comparators. Three of these studies were rated as high quality, whereas one was rated as fair quality in accordance with the PEDro scale ( Table 3). These studies commonly failed to meet criteria regarding allocation concealment, blinding of participants, therapists or assessors. Therefore, even though these studies were determined as adequate quality, there was a risk of selection as well as detection bias, which may lead to significant alteration in the findings.

Other limitation of these studies was that they most commonly used subjective outcome measures such as Vertigo Symptom Scale, VAS, and Short Form-36. Nonetheless, the objective outcome measures were significant to ensure reliable rationale for treatment. Furthermore, they may demonstrate significant limitations quantifying objectively. On the other hand, subjective outcome measures are based on the perception of the individual with regards to the symptoms. The Hall *et al.* ^18^ study contained a very small cohort (n = 37), which makes it difficult to generalise the results to a wide range of dizzy patients.

The effectiveness of VR in dizziness has been currently evaluated by several studies ^11, 22, 23^. However, previous systematic reviews evaluated the effect of VR very generally ^11, 23^ or focused on only passive repositioning manoeuvres ^22^. For instance, a SR conducted by Kendall *et al.* ^23^ evaluated all non-pharmacological interventions and did not focus on only VR, while Ricci *et al.* ^11^ studied the effectiveness of VR for all vestibular problems and dizziness in general. Given the lack of targeted studies in this area, this SR aimed to critically appraise the existing evidence and provide further information regarding the effectiveness of exercise-based VR to tackle chronic dizziness in adult patients. To the authors’ knowledge, this study is the first SR investigating the effectiveness of exercise-based VR for chronic dizziness in adult patients.

The authors’ acknowledge several weaknesses which exist within this SR. Firstly, five databases were investigated to identify relevant literature regarding VR and the research process did not include grey literature. Therefore, it is possible some relevant studies were overlooked. Secondly, studies published in languages other than English were excluded, but there might have been some relevant and valuable studies in those non-English articles (language bias). Finally, studies in this SR generally compared the effectiveness of VR interventions that were nurse-delivered or booklet-based, without supervision, other types of VR protocols may not have been represented in the literature. It may have been preferable to compare the same delivery method or similar exercise protocols. Consequently, there is a need for future studies, including homogenous samples.

The subjective measurement of symptoms was the most commonly used outcome measure in all the included studies. Three of the studies ^19– 21^ used the Vertigo Symptom Scale, whereas one study ^18^ assessed the alteration in symptoms using the Disability Rating Scale and the VAS. Yardley *et al.* ^21^ reported that the relative risk of the improvement in symptoms of the VR group versus the usual medical care group was 1.78 (95% CI, 1.31 to 2.42), while 56 out of 83 participants (67%) in the VR group reported easing in symptoms, in the usual medical care group it was only reported by 33 of 87 patients (38%) at the three-month follow-up.

Hall *et al.* ^18^ were unable to find significant difference between the gaze stabilisation exercise group and control group to whom placebo eye exercises were applied, except for fall risk measured by Dynamic Gait Index (DGI) (p = 0.026). However, in this study, the paucity of the number of participants (n = 37) and the compliance of patients make the findings questionable. Firstly, the number of patients was limited in this study. Secondly, patients in the control group were compliant with the placebo eye exercises eighty percent of the time, while in the gaze stabilisation group, the compliance of the patients was sixty percent of the time (p = 0.04). This result is consistent with the study of Shumway-Cook *et al.* ^24^, which concluded that patients who are compliant with the therapy sessions less than 75% of the time demonstrated less improvement compared the patients who are fully adherent to therapy. Last, this difference in the findings might arise due to the duration of experiment. Participants were assessed at baseline and at discharge, which was only 2–3 weeks for many participants. Likewise, another included study by Yardley *et al.* ^19^ failed to reach significance at 12 weeks, while it found a clear improvement compared to the routine care group at one year follow-up. Therefore, long-term outcomes might be more effective than short-term ones.

Another outcome measure was the postural control evaluated by using static (Romberg Test) ^20, 21^ and dynamic (DGI) ^18^ balance tests and computerised dynamic posturography ^18^. Although the static balance tests are easy to apply, they are deficient in the evaluation of functional aspects of balance and mobility. Functional balance tests may complete these aspects providing information regarding characteristics, gait and balance. However, it is also limited in determining muscle tightness or weakness and motor coordination problems, which are important aspects of an individualised treatment plan. Conversely, computerised dynamic posturography might assess all of these signs and is useful for determining the diagnosis of vestibular disorder and an appropriate treatment plan to complete other conventional balance tests.

## Conclusion

This review suggests that exercise-based VR shows benefits for adult patients with chronic dizziness in terms of improvement in the vertigo symptom scale, fall risk, balance and emotional status. Three out of four studies demonstrated a significant difference in the VR group compared to the usual medical care group. Furthermore, it was also suggested that VR is cost-effective, either by direct delivery in primary care units or via a booklet-based delivery. However, this study could not determine the most effective VR protocol or the optimal treatment frequency and intensity, so further studies are required to elucidate the optimal methods of delivery and dosages.

## Data availability

*All data underlying the results are available as part of the article and no additional source data are required*

## Supplementary material

Supplementary File 1: Completed PRISMA checklist.
