# Supplementary material for: The effectiveness of exercise-based vestibular rehabilitation in adult patients with chronic dizziness: A systematic review
Source: F1000Res. 2018 Mar 5;7:276. [Version 1] doi: 10.12688/f1000research.14089.1 (PMC5954334; doi:10.12688/f1000research.14089.1)
Supplement: Peer review history

# Peer review: Referee response for version 1

**DOI:** 10.5256/f1000research.15325.r33648

**Published:** 2018-05-14

**Authors:** Alia Alghwiri

**Affiliations:** 1 Department of Physical Therapy, Faculty of Rehabilitation Sciences, The University of Jordan, Amman, Jordan

This systematic review is an interesting paper that highlights the effectiveness of exercise-based vestibular rehabilitation in adult patients with chronic dizziness.

 The rationale behind conducting this paper is clear.

 The methods section has sufficient and clear details.

 The results of the study was stated clearly and a summary of the findings was provided in a good way.

 The discussion and conclusion provide adequate information that is supported by the results.

I have read this submission. I believe that I have an appropriate level of expertise to confirm that it is of an acceptable scientific standard.

# Peer review: Referee response for version 1

**DOI:** 10.5256/f1000research.15325.r31549

**Published:** 2018-03-16

**Authors:** Shamekh Mohamed El-Shamy

**Affiliations:** 1 Department of Physical Therapy for Pediatrics, Faculty of Physical Therapy, Cairo University, Giza, Egypt

Thank you for the invitation to review this paper.

 General comment:

 This paper aimed ‘to investigate the effectiveness of exercise-based vestibular rehabilitation in adult patients with chronic dizziness.' Generally, the manuscript content was well-written. The introduction section was concise and clear. The methods and results sections were well-structured. The discussion section was well-explained.

 Specific comments:

 Strengths:

1. Introduction section: Generally, the introduction section covered all necessary data, including: definition of dizziness, epidemiology, impact of dizziness on society, classifications of dizziness and their definitions, etiologies of dizziness and its symptoms, brief summary about available interventions, vestibular rehabilitation, the importance of this review and the aim.
2. Methods section: Reporting the data sources, an example of search strategy in detail that was presented in table 1 which showed the specific terms that used in the search process, inclusion and exclusion criteria, data extraction, risk of bias assessment, data synthesis and analysis.
3. Result section: Study selection and characteristics were reported clearly, risk of bias assessment, and reporting the results and divided them based on the outcome measures which was easy to read and follow.
4. Discussion section: interpretation of the results and reporting some of the limitations of the study.

Weaknesses:

1. Grey literature was not included in the search process.
2. The authors did not report the effect size for each study to determine the effectiveness level of the intervention.
3. The authors assessed the risk of bias for each included study, but they did not report the overall quality of a body of evidence.
4. The discussion section did not include enough supporting evidence.

Generally, although the existence of these weaknesses, there are no necessary changes to the content of the manuscript.

I have read this submission. I believe that I have an appropriate level of expertise to confirm that it is of an acceptable scientific standard.
